# The Hemodynamic Basis for Positional- and Inter-Fetal Dependent Effects in Dual Arterial Supply of Mouse Pregnancies

**DOI:** 10.1371/journal.pone.0052273

**Published:** 2012-12-20

**Authors:** Tal Raz, Reut Avni, Yoseph Addadi, Yoni Cohen, Ariel J. Jaffa, Brian Hemmings, Joel R. Garbow, Michal Neeman

**Affiliations:** 1 Department of Biological Regulation, Weizmann Institute of Science, Rehovot, Israel; 2 Lis Maternity Hospital, Tel Aviv Souraski Medical Center, Tel Aviv, Israel; 3 Friedrich Miescher Institute for Biomedical Research, Basel, Switzerland; 4 Biomedical MR Laboratory, Mallinckrodt Institute of Radiology, Washington University, St. Louis, Missouri, United States of America; Otto-von-Guericke University Magdeburg, Germany

## Abstract

In mammalian pregnancy, maternal cardiovascular adaptations must match the requirements of the growing fetus(es), and respond to physiologic and pathologic conditions. Such adaptations are particularly demanding for mammals bearing large-litter pregnancies, with their inherent conflict between the interests of each individual fetus and the welfare of the entire progeny. The mouse is the most common animal model used to study development and genetics, as well as pregnancy-related diseases. Previous studies suggested that in mice, maternal blood flow to the placentas occurs via a single arterial uterine loop generated by arterial-arterial anastomosis of the uterine artery to the uterine branch of the ovarian artery, resulting in counter bi-directional blood flow. However, we provide here experimental evidence that each placenta is actually supplied by two distinct arterial inputs stemming from the uterine artery and from the uterine branch of the ovarian artery, with position-dependent contribution of flow from each source. Moreover, we report significant positional- and inter-fetal dependent alteration of placental perfusion, which were detected by in vivo MRI and fluorescence imaging. Maternal blood flow to the placentas was dependent on litter size and was attenuated for placentas located centrally along the uterine horn. Distinctive apposing, inter-fetal hemodynamic effects of either reduced or elevated maternal blood flow, were measured for placenta of normal fetuses that are positioned adjacent to either pathological, or to hypovascular *Akt1*-deficient placentas, respectively. The results reported here underscore the critical importance of confounding local and systemic in utero effects on phenotype presentation, in general and in the setting of genetically modified mice. The unique robustness and plasticity of the uterine vasculature architecture, as reported in this study, can explain the ability to accommodate varying litter sizes, sustain large-litter pregnancies and overcome pathologic challenges. Remarkably, the dual arterial supply is evolutionary conserved in mammals bearing a single offspring, including primates.

## Introduction

Pregnancy in mammals requires coordinated adaptation of the systemic maternal cardiovascular system and remodeling of the entire uterine circulation, to assure adequate oxygenation and nutrient delivery to the developing embryo/fetus [1]–[3]. A variety of prenatal syndromes in humans, including intrauterine growth restriction (IUGR), preeclampsia, and fetal death in utero have been attributed to insufficient placental perfusion. Maternal vascular adaptation to pregnancy includes vascular remodeling, increased uterine blood flow, and systemic increase in total blood volume and cardiac output [1], [4]–[8]. The consequences of placental malfunction include predisposition to systemic cardiovascular and pulmonary vascular dysfunction [9]–[12] associated with increased risk for ischaemic heart disease throughout life [13], [14].

To insure successful reproduction, mammals have adopted one of two primary strategies. In some species, including humans, non-human primates and horses, single-fetus pregnancy was optimized for high survival rate. The alternative strategy, found for example in porcine, canines, felines and rodents, is delivery of large litters. To achieve large successful litters, the reproductive system must be optimized in the face of an inherent conflict between the benefit to each individual fetus and the benefit to the species. An important example of large-litter pregnancy is the mouse. The uterus of the mouse is duplex, composed of two independent uterine horns that converge at the cervix [15]. Blood enters the mouse uterus cranially through the uterine branch of the ovarian artery and caudally through the uterine artery [15], [16]. Studies of McLaren and Michie in the late 1950s reported that pregnancy is supported in each of the two uterine horns by a single uterine arterial loop, which is generated by direct artery-to-artery anastomosis of the uterine artery with the uterine branch of the ovarian artery [15]–[17]. The direction of blood flow in the single uterine arterial loop was described as being dominated by the uterine branch of the ovarian artery in the cranial portion of each horn, and by the distal part of the uterine artery on the caudal end. Such counter flow within this single loop was postulated to result in a significant drop in pressure and flow velocity to the central embryos/fetuses in each uterine horn [16], [17]. We argue that such an anatomical structure, including opposing flow within a single arterial loop, results in a hemodynamic enigma, in which the central fetuses receive little or even zero perfusion. This appears to be an unfavorable structure for maintaining stable and robust distribution of perfusion between placentas in a large litter, as the middle fetuses may not survive the course pregnancy. Moreover, opposing flow within a single arterial loop is unreasonable in terms of the hemodynamics of blood flow.

To evaluate the anatomy and function of maternal circulation to support pregnancy in the mouse, we adopted state-of-the-art, non-invasive imaging tools. Over the last years, there has been significant progress in non-invasive imaging of mouse pregnancy [18]–[24]. These studies have focused on imaging anatomical details in developing embryos [25], [26] by ultrasound and MRI [22], and, in particular, analysis of the fetal heart and brain [27]–[30]. In addition, using ferritin as a reporter gene for MRI, we reported in vivo identification of fetal vascular development through the expression of VE-cadherin [31]. Furthermore, a number of studies investigated perfusion of the placenta in mice using contrast media such as GdDTPA and biotin-BSA-GdDTPA [19], [24], [32]. Of particular interest was the use of MRI for analysis of fetal development in *Akt1*-deficient mice. *Akt1*, also known as PKB alpha, is a major component of growth-factor-induced signaling, and was found to play a critical role in angiogenesis, cell survival and differentiation, and glucose metabolism [33]. *Akt1* deficiency leads to in utero growth retardation and death associated with deficiencies in placental vascularity [18], [34]–[37]; therefore, *Akt1* deficient mice have been suggested as a model for human IUGR [18], [38].

One of the complications of longitudinal studies of mouse pregnancy is the accurate identification of embryos in utero. We recently introduced the use of bi-directional arterial spin labeling MRI (BD-ASL MRI) for identification of fetal order along the uterus horn in vivo, based upon the specific pattern of the dual arterial blood supply within the mouse uterine horns [39]. Peak BD-ASL values showed highly significant positive correlation to the position of placentas along the uterine horn: peak BD-ASL values were found to be negative for placentas near the cervix and increased gradually for the middle placentas, with the placenta closest to the ovary having the largest positive signal. This ability to non-invasively determine fetus location along the uterine horn opened possibilities for determining and pursuing phenotypic alterations in genetic, as well as developmental, longitudinal studies.

The aim of the current study was to examine the hemodynamics of maternal circulation in mice, and to evaluate how it balances between the needs of individual fetuses and the entire litter to guarantee successful pregnancy. The anatomical description, and particularly the concept of bidirectional flow within a single uterine arterial loop, was revisited, using state-of-the-art imaging methods. We further analyzed the patterns of maternal-to-fetal water exchange and maternal blood volume in placentas during normal pregnancy, as well as in pregnancies complicated by spontaneous missed abortions, surgical ligation of specific arterial input, and genetic in utero growth retardation due to thymoma viral proto-oncogene 1 (*Akt1*) deficiency [18], [34]–[36].

## Materials and Methods

### Animal Models

**Figure 1:**
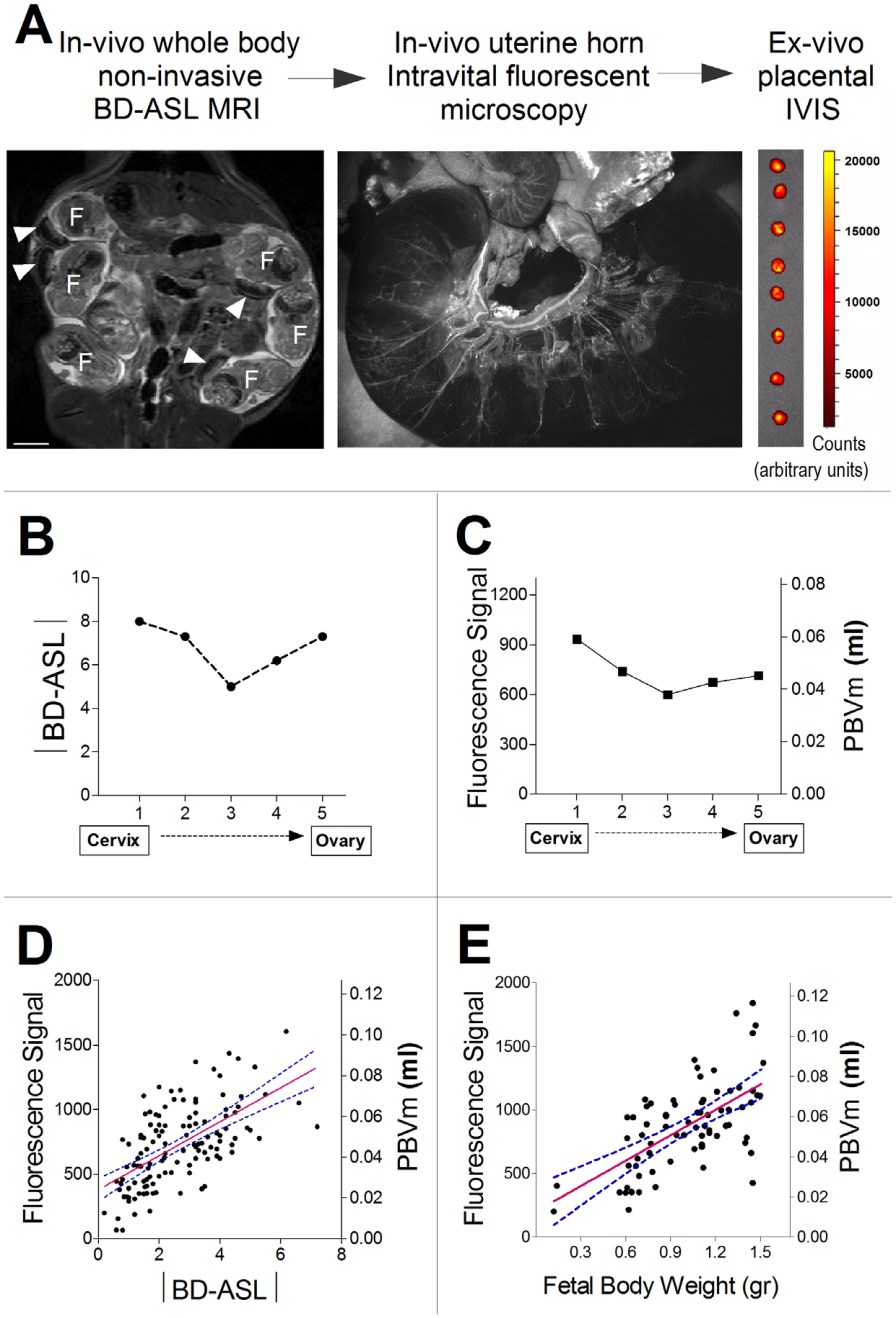
Position dependence of maternal blood to placentas along the uterine horn. (A) Experimental scheme for multi-modal functional imaging of pregnant mice: pregnant female ICR mice (E17.5) were analyzed using MRI, intravital fluorescence microscopy, and ex vivo fluorescence analysis of the maternal blood volume in the placenta (PBVm). Note fetuses (F) and their placentas (white arrow heads). (B) Data for a pregnant mouse carrying 5 fetuses in one uterine horn. Position dependence of maternal bi-directional perfusion was detected by MRI (|BD-ASL|). (C) Ex vivo fluorescence and corresponding PBVm values for the placentas in one uterine horn, in the same pregnant mouse as in A. (D) Correlation between PBVm and |BD-ASL| (n = 11 dams, 86 placentas/fetuses; r = 0.62, *P*<0.0001). (E) Correlation between PBVm and fetal body weight (n = 11 dams, 86 placentas/fetuses; r = 0.64, *P*<0.0001).

All experiments were approved by the Institutional Animal Care and Use Committee (IACUC) of the Weizmann Institute of Science (Permit Number: 03020610-2). Female ICR mice (n = 11 dams, 86 placentas/fetuses) and B6(C57BL/6J)/*Akt1^+/−^*mice (n = 17 dams, 128 fetuses/placentas) were analyzed on embryonic day E17.5 (E0.5 =  day of vaginal plug, which indicates mating). Multimodal imaging consisted of: 1) in vivo, whole body, non-invasive MR imaging, including fast spin-echo MRI followed by BD-ASL MRI [39], 2) in vivo intravital microscopy of surgically-exposed uterine horns following intravenous administration of fluorescent FITC-dextran, and 3) ex vivo FITC-dextran fluorescence analysis of the maternal blood volume in the same placentas (Fig. 1).

### In Vivo MRI Studies

MRI experiments were performed at 9.4T on a BioSpec 94/20 USR spectrometer (Bruker; Karlsruhe, Germany). A quadrature volume coil (72 mm i.d.) was used for both transmit and receive. Anesthetized mice (isoflurane, 3% for induction, 1–2% for maintenance; Abbott Laboratories Ltd, England; in oxygen,1 liter/min delivered through a muzzle mask) were placed in a supine position, with respiratory and thermal monitoring (SA Instruments Model 1025 monitoring and gating system, Stony Brook, NY, USA; 50–70 breaths per min; 37°C body temperature, maintained using a circulating water system).

Fast spin-echo MRI using a RARE sequence (Rapid Acquisition with Refocused Echoes) was used to visualize all feto-placental units using the following parameters: effective echo time (TE) = 85.5 ms; RARE factor = 8; slice thickness 1.0 mm; intersection gap 0.2 mm; matrix 256×128, zero filled to 256×256; coronal: repetition time (TR) = 3000 ms; field of view (FOV) = 7×5 cm^2^; axial: TR = 4000 ms; FOV = 5×5 cm^2^; 15–40 slices were acquired to cover all the feto-placental units.

Placental perfusion was measured using Bi-Directional Arterial Spin Labeling data (BD-ASL), as described by Avni, et al. [39]. Briefly, multi-slice, axial two-dimensional gradient echo (2D–GE) data were acquired with the following parameters: TR = 53 ms; TE = 3.2 ms; slice thickness = 1.0 mm; intersection gap 0.2 mm; 4 slices per scan; FOV = 5×5 cm^2^; matrix = 256×128, zero filled to 256×256; 10 dummy scans; 32 averages; and a scan time with respiratory gating ∼6 min). A thick axial slice (8 mm) was used for saturation of blood in either the maternal heart or the bifurcation point of the common iliac artery (90° hermit pulse, 2 ms followed by a crusher gradient). BD-ASL data were analyzed with in-house programs written in Matlab (Mathworks; Natick, MA, USA), using the normalized percentile difference between the two acquired images:
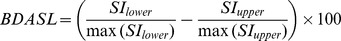
where SI_upper_ and SI_lower_ are the magnetization in the perfused organ with upper and lower steady-state saturation pulses, respectively [39]. The resulting analyses were used to produce saturation transfer BD-ASL maps, in which a change in signal intensities revealed the directional contribution of water exchange between the maternal blood and the placenta; accordingly, the signal in each placenta was color-coded (as blue or red pixels) according to its blood origin (i.e., uterine artery, or uterine branch of the ovarian artery, respectively). Placenta BD-ASL values depend on the contribution of the arterial supply direction: negative values represent placentas that are perfused more dominantly by the uterine artery, and positive values represent placentas that are nourished more dominantly by the uterine branch of the ovarian artery [39]. The absolute value of BD-ASL is proportional to tissue perfusion, namely the exchange of water across the labyrinth within the placenta.

### Fluorescence Analysis of the Uterine Blood Supply

The uterine blood supply was imaged using a Zoom Stereo Microscope SZX-RFL-2 (Olympus, Tokyo, Japan) equipped with a fluorescence illuminator and a Pixelfly camera (PCO, Kelheim Germany), and analyzed using ImageJ (http://rsbweb.nih.gov/ij/). Mice were anesthetized with Ketamin (i.p.; Ketaset®, Fort Dodge Laboratories, Fort Dodge, IA, USA; 100 mg/kg) and Xylazine (i.p.; XYL-M, VMD, Arendonk, Belgium; 16 mg/kg). The uterus was surgically exposed through a ventral midline celiotomy and the two uterine horns were spread out to expose the intact blood vessels. Dextran–fluorescein isothiocyanate (6 mg diluted in 300 µl PBS per animal; FITC, 500 kDa; Sigma Aldrich) was injected via a tail-vein catheter for acquisition of dynamic data (1200 frames/min for 0.5 min; exposure time of 50 ms; excitation 460 – 490 nm; emission 510 – 550 nm). Preliminary experiments revealed an equilibration time of 10 minutes in order to reach a steady-state fluorescent signal in the placental vasculature. Thus, the reported fluorescent intensity was acquired 10 minutes after administration of the contrast media. Signal enhancement was normalized for each placenta, using the pre-injection value.

Ex vivo fluorescence studies of maternal blood volume in the placentas were performed using an IVIS 100 Imaging System (Xenogen; Cranbury, NJ, USA; excitation 500 nm; emission 540 nm). Placental fluorescence was normalized to the intra-vital signal measured in the uterine artery 10 minutes post FITC-dextran administration.

Placental maternal blood volume (PBVm; in ml) was derived using calibration measurements on blood samples from ICR female mice 10 min after intravenous dextran-FITC administration (n = 6; E17.5). The fluorescence of blood samples (0.01, 0.03, 0.05, 0.08, 0.1, 0.15, 0.3 ml; IVIS), normalized to the intravital fluorescence in the uterine artery, was positively correlated to the blood volume (r = 0.97, *P*<0.0001; Fig. S1). Using this calibration, the estimated PBVm in the examined placentas of this group of mice (mean±SEM) was calculated to be 0.072±0.002 ml.

### Ligation of Arterial Blood Supply to the Uterine Horn

Arterial ligations were performed on anesthetized pregnant ICR mice (E17.5, n = 20). The uterus was surgically exposed through a ventral midline celiotomy. Surgical ligations were performed in either the uterine branch of the ovarian artery (n = 10 mice, 5 mice on each side; 82 placentas/fetuses), or in the uterine artery (n = 10 mice, 5 mice on each side; 92 placentas/fetuses), using a Surgeon Knot (PDS 5–0 suture material, Ethicon, San Angelo, TX, USA). Immediately after the ligation, mice were positioned for fluorescence intravital imaging using dextran-FITC administration, as described above. The fluorescence signal in each placenta in the ligated side was calibrated to the average fluorescence of all placentas in the control side. The relative contribution of each ligated artery to the overall placental blood volume in the horn, in each animal, was calculated according to the following equation:
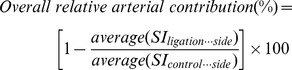



The position-dependent contribution of each artery to each placenta was calculated using the data obtained only from those animals which had a symmetric pregnancy (either 4 fetuses/placentas in each uterine horn, n = 5 dams; or 6 fetuses/placentas in each uterine horn, n = 4 dams), according to the following equation:
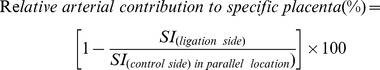
where SI(ligation or control side) is the fluorescence signal in a placenta located in the same position along the uterine horn in the ligated or control side, respectively.

### Validation of Fetal Location and Genotype

For all animals, the positions of the uterus and all fetuses/placentas were recorded by digital camera during ventral midline celiotomy surgery, as described above, or following euthanasia by cervical dislocation. In B6(C57BL/6J)/*Akt1^+/−^* mice, a sample was taken from the tail of each fetus for genotyping when placentas were obtained for ex vivo fluorescence analysis.

### Histology and Fluorescence Microscopy

Placentas were fixed in Carnoy solution, embedded in paraffin, sectioned serially (4 µm thickness), and stained with hematoxylin and eosin (H&E; Sigma-Aldrich) or with 4′,6-diamidino-2-phenylindole (DAPI; Vector Laboratories, Burlingame, CA, USA). Images were obtained with a Zeiss Axio observer microscope (Yena, Germany) equipped with a fluorescence illuminator and an Olympus DP72 camera (Center Valley, PA, USA; FITC, 440 – 470 nm excitation and 525 – 550 nm emission; DAPI, 365 nm excitation and 445 – 450 nm emission).

### Statistical Analysis of the Experimental Data

Statistical analyses were performed with analytic computerized software (Statistix 8 Student Edition, Analytical Software, Tallahassee, FL, USA). The general effects of placental location, litter size, and fetal body weights on BD-ASL and fluorescent measurements (intravital microscopy or IVIS data) were analyzed in a General Analysis of Variance Test (ANOVA), followed by Tukey HSD All-Pairwise Comparisons test. Continuous data were evaluated for normality of distribution and for equality of variances using the Shapiro-Wilk Test and the Bartlett’s Test, respectively. Pearson’s Correlation Test was used to determine various correlations between BD-ASL, ex vivo placental fluorescence signal, placenta location, litter size, and fetal body weight. Pearson Chi-square Test analysis was used to compare proportional data. Differences were considered significant at *P*<0.05. Unless otherwise noted, results are presented as mean ± SEM.

### Modeling the Pattern of Arterial Blood Supply to the Uterine Horns by Electrical Circuit

Maternal circulation in the uterus was modeled as an electrical circuit, with maternal cardiac output simulated by a single, constant voltage (V_H_) battery. The current in each placenta was derived numerically using a computer simulation program, PSPICE 9.1 (Cadence, http://www.cadence.com), with simulations based upon standard KCL (Kirchhoff current law) and KVL (Kirchhoff voltage law) analysis.

## Results

### Maternal Blood Volume and Exchange are Attenuated for Placenta Located Centrally along the Uterine Horn

To evaluate the maternal-to-placental arterial exchange and placental maternal blood volume, a multi-modality imaging approach was applied in ICR pregnant mice on embryonic day E17.5. The multimodal imaging consisted of MRI, intravital microscopy, and fluorescence analysis of the maternal blood volume in the same placentas (PBVm; Fig. 1A–C). Peak BD-ASL values (n = 11 dams; 83 placentas/fetuses) were consistent with fetal position along the uterine horn, as reported previously [39]. Absolute BD-ASL values (|BD-ASL|), corresponding to maternal-fetal arterial water exchange, were significantly larger for the placentas that were positioned at the two extremes of the horns, compared to those in the middle (*P* = 0.0034). Furthermore, |BD-ASL| values were significantly larger for placentas adjacent to the cervix, compared to those closer to the ovary (*P* = 0.0107).

Within animals, placental fluorescence values and corresponding PBVm displayed a U-shaped pattern, with larger values of enhancements for the placentas located near the origin of blood vessels (*P*<0.02; Fig. 1B, 1C). Overall, PBVm was positively correlated with fetal body weight (Fig. 1E; r = 0.64, *P*<0.0001). |BD-ASL| values (maternal-to-placental arterial exchange) significantly correlated with placental FITC-dextran fluorescence (PBVm; Fig. 1D; r = 0.62, *P*<0.0001).

### Effects of Litter Size and the Position along the Uterine Horn on Placental Perfusion

**Figure 2:**
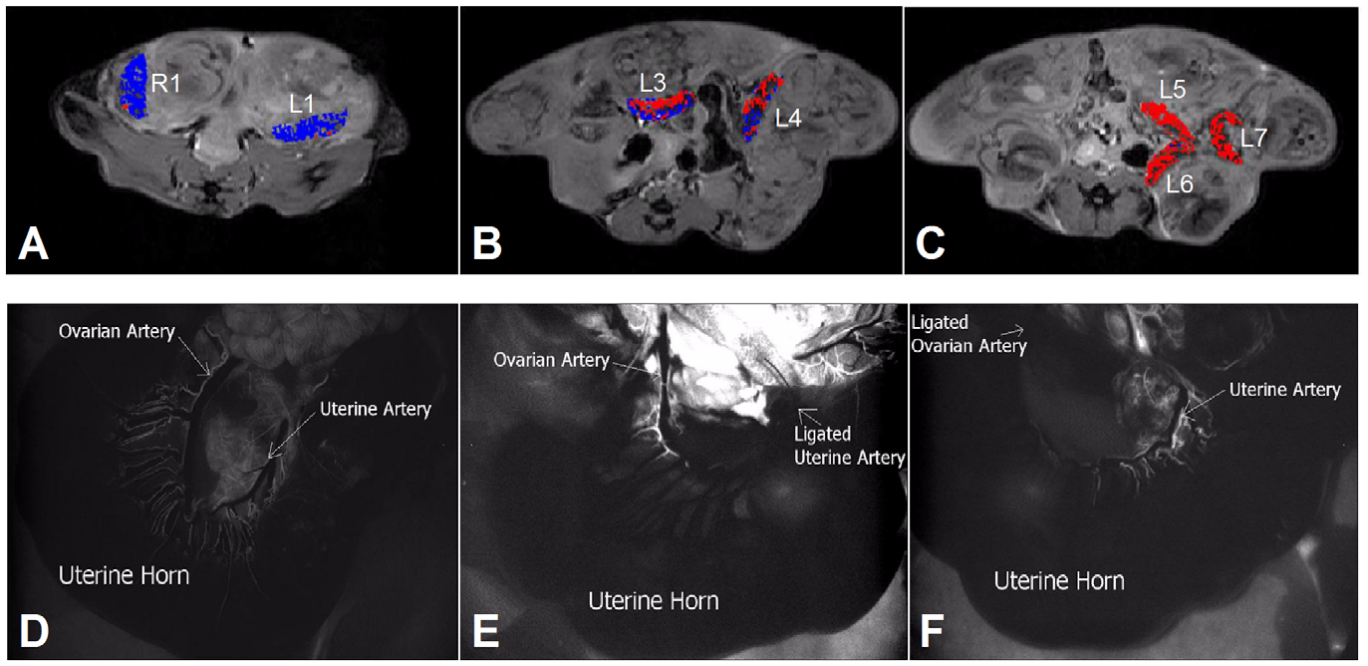
Assessment of the arterial blood supply to the placenta via BD-ASL MRI and intravital fluorescence microscopy. Two methods were used to explore the pattern of transfer of arterial blood to the placentas along the uterine horns in pregnant mice at late gestation (E17.5): 1) Bi-directional ASL methodology (Panels A–C); and 2) Intravital fluorescence microscopy imaging of the uterine arterial blood supply subsequent to intravenous administration of FITC-dextran to mice having undergone surgical arterial ligations of either the uterine branch of the ovarian artery, or the uterine artery (Panels D–F). (A–C) Placental saturation transfer maps obtained by BD-ASL MRI of an ICR pregnant mouse (E17.5). For placentas positioned closer to the cervix (Panel A: L1, R1), mainly negative BD-ASL contrast voxels (blue) were observed, consistent with the predominant contribution of maternal blood flow through the uterine artery. In placentas closer to the ovary (Panel C: L5–7), the BD-ASL contrast was mainly of positive voxels (red), implying that placentas in this part of the uterine horn are supplied through blood mainly from the uterine branch of the ovarian artery. Placentas located in the central region of the uterine horn (Panel B, L3–4) had a dispersive pattern of BD-ASL values with both negative and positive voxels, consistent with a dual supply from both the uterine artery and the uterine branch of the ovarian artery, respectively. (D) Intravital fluorescence microscopy image of the arterial blood supply to an intact uterine horn (a snapshot from Movie S1). (E) Intravital fluorescence microscopy image of the arterial blood supply to a uterine horn following ligation of the uterine artery (a snapshot from Movie S2). (F) Intravital fluorescence microscopy image of the arterial blood supply to a uterine horn following ligation of the uterine branch of the ovarian artery (a snapshot from Movie S3).

Bi-directional ASL methodology was employed to explore the pattern of transfer of saturated blood to the placentas along the uterine horns in pregnant mice at late gestation. For placentas positioned closer to the cervix, mainly negative BD-ASL contrast voxels were observed (Fig. 2A, blue), consistent with the predominant contribution of maternal blood flow through the uterine artery. In placentas closer to the ovary, the BD-ASL contrast consisted mainly of positive voxels (Fig. 2C), indicating that in the cranial portion of the uterine horn, the major blood supply is through the uterine branch of the ovarian artery. Interestingly, for placentas located in the central region of particularly crowded horns, the BD-ASL contrast inside the placenta included both negative (Fig. 2B, blue) and positive (Fig. 2B, red) voxels, consistent with a dual supply from both the uterine artery and the uterine branch of the ovarian artery, respectively. Moreover, for all placentas, maternal arterial input was consistent with distinct irrigation fields for each arterial input, while inconsistent with a single mixed arterial input, as would be expected for anastomosis of the two arteries.

In order to determine the selective contribution of each arterial blood supply to the uterine horn, selective surgical arterial ligations (either the uterine artery, or the uterine branch of the ovarian artery) were performed on anesthetized ICR pregnant mice (E17.5); the exposed intact uterine horns were imaged by fluorescence intravital microscopy following dextran-FITC administration, while the arterial blood vessels to the uterine horns and the placentas were imaged (Fig. 2D–F, represent Movies S1, S2, and S3 for non-ligated control, ligation of the uterine artery, and ligation of the uterine branch of ovarian artery, respectively). Thereafter, the ex vivo fluorescence signals in all placentas were measured, as described in Methods. The type of ligation (uterine artery vs. uterine branch of ovarian artery) had a significant effect on the relative FITC-dextran fluorescence in the corresponding placentas, as well as on the signal dynamics in the major arteries supplying the uterine horn (*P*<0.0001).

**Figure 3:**
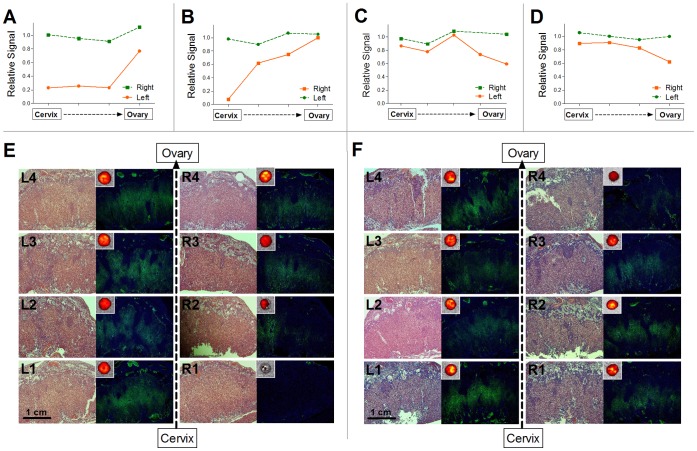
Contribution of arterial blood supply to the placenta. Arterial ligations were performed on ICR pregnant mice (E17.5) for either the uterine branch of the ovarian artery (n = 10 mice, 5 mice on each side; 82 placentas/fetuses); or in the uterine artery (n = 10 mice, 5 mice on each side; 92 placentas/fetuses). (A, B) Relative fluorescence signal in placentas along two uterine horns upon ligation of the left (A) or the right (B) uterine artery (each graph presents data from one animal. orange: ligated uterine horn; green: non-ligated uterine horn). (C, D) Relative fluorescence signal in placentas along two uterine horns upon ligation of the left (C) or right (D) ovarian artery (each graph presents data from one animal). (E, F) Histological sections of the placentas along two uterine horns upon ligation of the right uterine artery (E; same animal as in panel B) or right ovarian artery (F; same animal as in panel (D) Left) H&E section. Right) fluorescence microscopy (right; Green = FITC-dextran; Blue =  DAPI; inset fluorescent image of the ex vivo placenta).

Ligation of the uterine artery caused a marked position-dependent reduction in the fluorescence signal in most of the placentas along the uterine horn, as compared to placentas along the non-ligated horn (for specific examples see Fig. 3A and 3B). Ligations of the uterine branch of the ovarian artery resulted in less remarkable changes in the fluorescence-signal intensities of the placentas (for specific examples see Fig. 3C and 3D); the placenta closest to the ligation point (nearest the ovary) displayed only moderate reduction in fluorescence, whereas placentas located near the cervix exhibited almost no reduction in signal. Histological analysis of the placentas demonstrated a decreasing gradient of fluorescent signal enhancements from the ligation points onwards (Fig. 3E and 3F).

**Figure 4:**
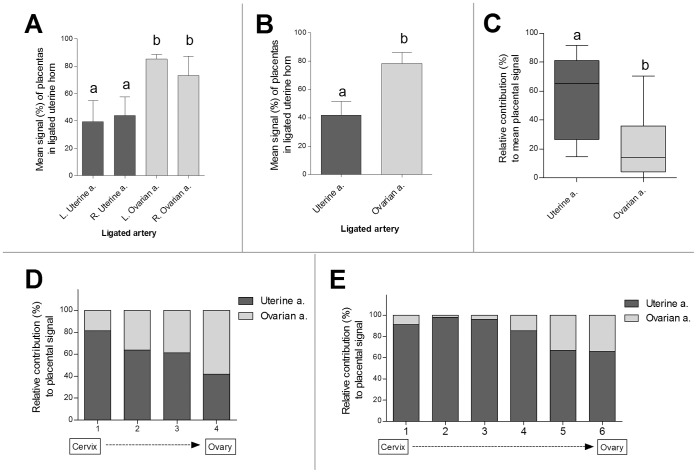
Arterial delivery of maternal blood to the placentas is dependent on litter size and on the position of the placenta along the uterine horn. (A) Mean fluorescence signal in placentas of a uterine horn after ligation of the left uterine a. (artery), right uterine a., left ovarian a., or right ovarian a. (n = 5 animal/group). For each animal, values were normalized (%) to the mean fluorescence signal of the placentas in the contralateral, non-ligated horn (mean ± SEM; a,b significant differences: *P*<0.05). (B) Mean fluorescence signal in placentas of a uterine horn after unilateral ligation of the uterine or ovarian arteries (n = 10 animal/group, left and right sides combined). For each animal, values were normalized (%) to the mean fluorescence signal of the placentas in the contralateral non-ligated horn (mean ± SEM; a,b significant differences: *P*<0.05). (C) Overall relative contributions of the uterine and ovarian arteries to the mean fluorescence signal in placentas in a uterine horn. Data are presented as Box-and-whisker plots, with the median, the 25% and 75% percentile ranges (box depth), and the maximum and minimum (T-bars). Significant differences (a, b; *P*<0.05). (D) Relative contributions of the uterine and ovarian arteries to the fluorescence signal in placentas located in different positions along the horn. Data were extracted only from symmetric pregnancies that contained 4 fetuses in each horn (n = 5 dams). (E) Relative contributions of the uterine and ovarian arteries to the fluorescence signal in placentas located in different positions along the horn. Data were extracted only from symmetric pregnancies that contained 6 fetuses in each horn (n = 4 dams).

The mean ex vivo placental fluorescence intensity in the ligated horn (relative to the mean signal in the non-ligated horn) was significantly affected by the type of ligation, with ligation of the uterine artery causing a larger reduction in signal compared to ligation of the uterine branch of the ovarian artery (Fig. 4A and 4B; *P*<0.0001). The side of the ligation (left vs. right; Fig. 4A) had no effect (uterine ligations: *P* = 0.758; uterine branch of ovarian artery: *P* = 0.43). The relative contribution of each artery to the fluorescence was calculated for the placentas along the uterine horn (mean [95% CI]). Overall, the uterine artery contributed 68% [55%–82%], while the uterine branch of the ovarian artery contributed 32% [18%–45%]. The contribution of each artery to each placenta was affected by litter size and the location of the placentas along the uterine horn (Fig. 4D and 4E).

### Intra-Uterine Neighbor Effect: Sibling Implanted Adjacent to a Pathological/Dead Fetus Exhibits Reduced |BD-ASL| and PBVm

**Figure 5:**
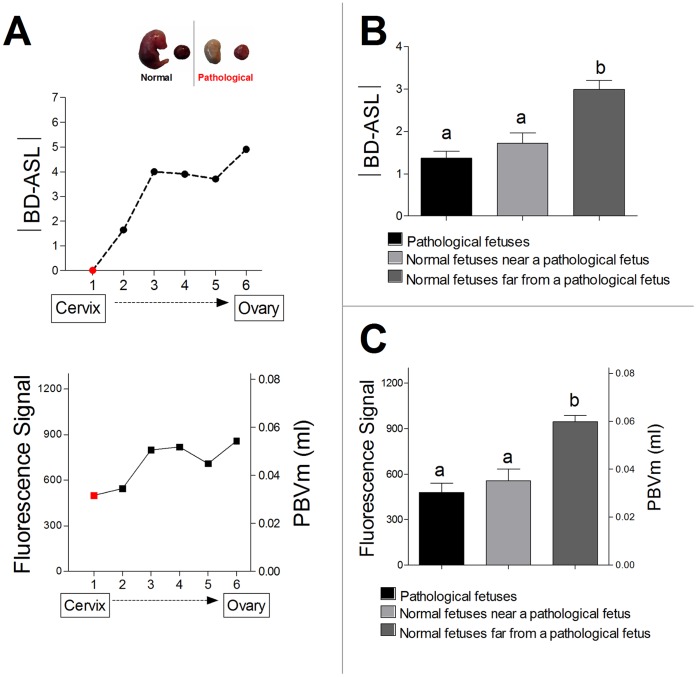
Intra-uterine neighbor effect: reduced |BD-ASL| and PBVm in placenta positioned adjacent to a pathological/dead fetus. (A) A representative example from one animal; placental |BD-ASL| (upper panel), and placental fluorescence signal with the corresponding PBVm values (lower panel) of placentas along a uterine horn that contains one pathological fetus (#1, marked in red). The upper image presents the pathological fetus and its placenta adjacent to a normal fetus and placenta (fetus #3). Note the reductions in |BD-ASL| and PBVm values in the placenta of the pathological fetus, as well as in the adjacent placenta (#2). (B) Mean |BD-ASL| values in placentas of pathological/dead fetuses, fetuses positioned adjacent to pathological/dead fetus, and normal fetuses located far from a pathological/dead fetus. Note that mean |BD-ASL| value is significantly lower for those fetuses that have a pathological/dead neighbor. Different letters above bars indicate significant differences (mean ± SEM; a,b, *P*<0.05) (C) Fluorescence values and corresponding PBVm values in placentas of pathological/dead fetuses, fetuses located near a pathological/dead fetus, and normal fetuses located distant from a pathological/dead fetus. Note that mean fluorescence value and PBVm values are significantly lower for those fetuses that have a pathological/dead neighbor (mean ± SEM; a,b, *P*<0.05).

In mice, as in any other litter-bearing mammals, the fetuses must share space in the uterus, suggesting that different fetuses might be exposed to different local environments. Litter size had a systemic effect on fetal body weight (r = −0.7838, *P*<0.0001). Larger litters were associated with higher incidence of severe fetal pathology/death (r = 0.6768, *P*<0.0001). Fetal death/pathology showed no positional dependence along the uterine horn (*P* = 0.6931). However, some effects were position dependent (Fig. 5); thus, beyond the expected low |BD-ASL| and PBVm values for placenta of pathological fetuses, the placenta located nearest to the pathological one also exhibited relatively low |BD-ASL| and PBVm values, compared with those predicted based on its location along the uterine horn (Fig. 5A).

To evaluate the impact of spontaneous fetal pathology/death on its neighbouring siblings in utero, all examined placentas (n = 11 mothers; 83 placentas/fetuses) were analyzed (Fig. 5B and 5C). Both |BD-ASL| and PBVm values of the placentas of pathological fetuses (|BD-ASL|: 1.3±0.1; PBVm: 0.030±0.003 ml), as well as their normal adjacent neighbours (|BD-ASL|: 1.7±0.2; PBVm: 0.035±0.004 ml), were significantly lower (*P*<0.008) compared to placentas lacking a neighbouring pathological fetus (|BD-ASL|: 3.0±0.2; PBVm: 0.060±0.002 ml). These results are consistent with a pathological/dead fetus having a significant negative influence on its most adjacent neighbouring sibling(s).

### Akt1^+/+^ Sibling Implanted Adjacent to an Akt1^−/−^ Placenta/Fetus Shows Increased |BD-ASL| and PBVm

**Figure 6:**
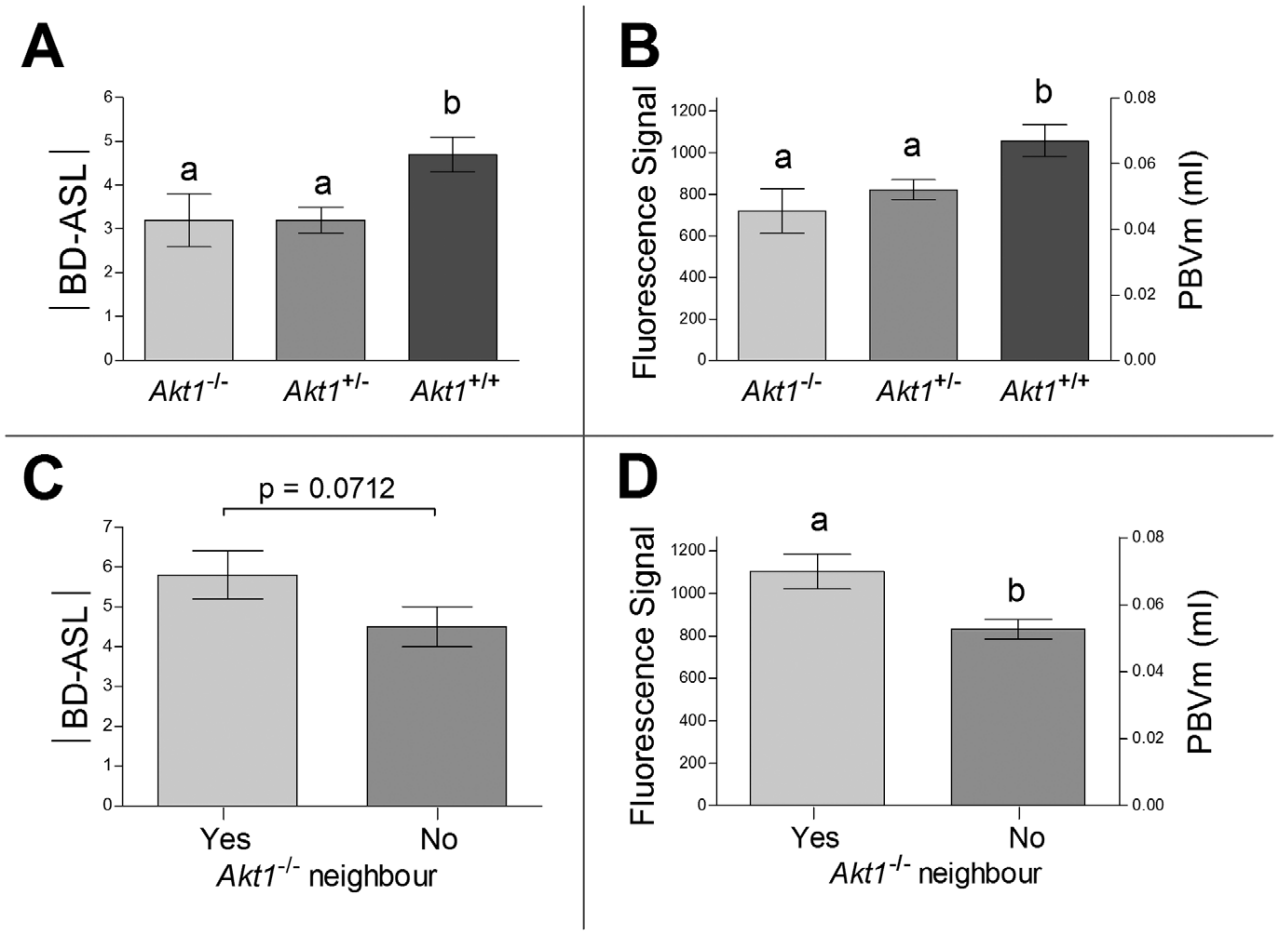
Placenta of *Akt1^+/+^* positioned adjacent to an *Akt1^−/−^* placenta/fetus showed increased |BD-ASL| and PBVm. (A) |BD-ASL| values in placentas of fetuses of different *Akt1* genotypes (mean ± SEM; a, b, *P*<0.05). (B) Fluorescence values and PBVm values in placentas of fetuses of different *Akt1* genotypes (mean ± SEM; a, b, *P*<0.05). (C) |BD-ASL| values in *Akt1^+/+^* placentas located near an *Akt1^−/−^* fetus, as compared to *Akt1* ^+/+^ placentas not located near an *Akt1^−/−^* fetus (mean ± SEM; *P* = 0.0712). (D) Fluorescence values and PBVm values in *Akt1^+/+^* placentas located near a *Akt1^−/−^* fetus, as compared to *Akt1^+/+^* placentas not located near an *Akt1^−/−^* fetus (mean ± SEM; a, b, *P*<0.05).

Thymoma viral proto-oncogene 1 (*Akt1*) plays an important role as an angiogenesis mediator and in placental vascular and cardiac function [18], [40]. It was shown previously that *Akt1^−/−^* placentas exhibited reduced maternal blood-volume fraction, as well as reduced area of circulatory bed and fetal growth retardation [18], [35], [36]. *Akt1^+/−^* female mice were mated with *Akt1^+/−^* males (n = 17 dams, 128 fetuses/placentas), to generate fetuses of different genetic backgrounds within the same pregnancy (*Akt1^+/+^*: 38 fetuses, *Akt1^+/−^*: 63 fetuses, *Akt1^−/−^*: 27 fetuses). All pregnant mice were analyzed by sequential imaging modalities, as described above. Fetal implantation location was found to be randomly distributed along the uterine horn for all genotypes (*P* = 0.8941). Placental peak BD-ASL values positively correlated with the relative location of fetuses along the uterine horn (r = 0.63, *P*<0.0001; Fig. S2). Consistent with the previously reported hypovascularity of Akt1^−/−^ placenta, placental |BD-ASL| and PBVm values (Fig. 6A and 6B) were significantly lower (*P*<0.02) for *Akt1^−/−^* placentas (|BD-ASL| = 3.2±0.6; PBVm: 0.045±0.006 ml) and *Akt1^+/−^* placentas (|BD-ASL| = 3.2±0.3; PBVm: 0.051±0.003 ml), compared to *Akt1^+/+^* placentas (|BD-ASL| = 4.7±0.4; PBVm: 0.067±0.004 ml).

We next evaluated the possible influence of *Akt1^−/−^* placentas on their adjacent *Akt1^+/+^* neighbors, and interestingly, found elevated PBVm values for *Akt1^+/+^* placentas that were positioned near *Akt1^−/−^* placentas (one or two) as compared to *Akt1^+/+^* placentas that were not near *Akt1^−/−^* placentas (Fig. 6D; 1257±141 vs. 792±71, respectively; *P* = 0.0177). |BD-ASL| values showed a similar trend (Fig. 6C; 5.8±0.6 vs. 4.5±0.5, respectively; *P* = 0.0712). Thus, healthy fetuses benefitted from having an *Akt1*-deficient near-neighbor sibling, in contradistinction to the detrimental neighbor effect of spontaneous pathologies.

### Simulating Placental Function in the Mouse Pregnancy as an Electrical Circuit Reveals a Possible Hemodynamic Basis for the Observed ‘Position Dependence’ and ‘Neighbor Effects’

**Figure 7:**
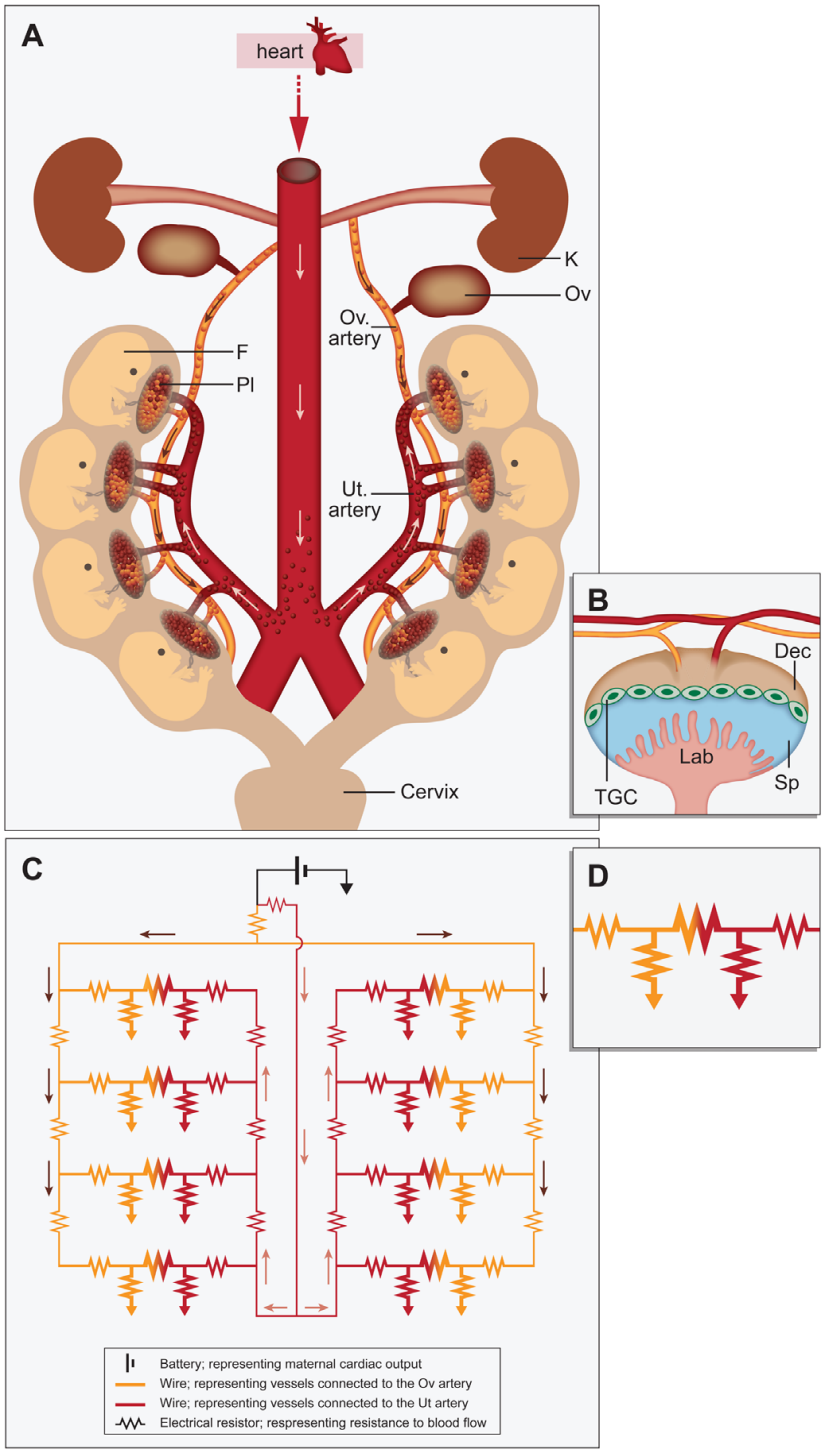
Electrical circuit modeling of the hemodynamics of maternal arterial supply in the mouse pregnancy. (A) Diagram of mouse uterine horns and their arterial blood vessels in the gestation period after placentas have formed (E10.5 to term). Our results demonstrate that each placenta can be perfused from two maternal arteries. F Fetus, K Kidney, Ov Ovary, Pl Placenta, Ut Uterine. (B) Schematic diagram of mouse placenta. Dec Decidua, Sp Spongiothrophoblast, TGC Throphoblast Giant Cells, Lab Labyrinth. (C) Numerical simulation of blood flow in multi-fetus pregnancy modeled as an electrical circuit. Bi-directional blood flow in each of two uterine horns was modeled by the respective currents: I_ua(l/r)_ for the left or right uterine arteries and I_oa(l/r)_ for the left or right uterine branch of the ovarian artery. The balance between I_ua(l/r)_ and I_oa(l/r)_ was set at 3∶1 using resistors placed into the circuit near the battery. Resistance to flow along the uterine branch of the ovarian artery and the uterine artery was modeled by a series of identical resistors (one per implantation site). (D) Placenta modeled as an electrical circuit. Resistance to flow into the placenta via the spiral arteries was modeled by low-value resistors, which were connected separately to a second resistor to ground, simulating exchange within the placenta itself and the flow back to ground (i.e., clearance of blood through the maternal veins). Diffusion across the placenta was modeled by the insertion of a large resistor (20× the value of the dual resistors to ground representing flow into the placenta), between the dual arterial input resistors. Total maternal blood supply to each placenta I_p,i_ was therefore derived from I_p,oa,i_+I_p,ua,i_. Arrows indicate the direction of flow.

Based on the experimental data reported here, we suggest a modified scheme for the functional anatomy of the mouse uterus, with its dual arterial blood supply, in late gestation (Fig. 7A, 7B). Specifically, each of the placentas can be nourished by branches from both arterial blood supplies (i.e., uterine artery and uterine branch of the ovarian artery), but in different proportions, depending on their positions along the uterine horn. The hemodynamics of such a vascular system was modeled by an electrical circuit (Fig. 7C, 7D).

The maternal heart was modeled as a battery (V_H_). Blood flow in each of two uterine horns was modeled by the respective currents: I_ua(l/r)_ for the left or right uterine arteries and I_oa(l/r)_ for the left or right uterine branch of the ovarian artery. The balance between I_ua(l/r)_ and I_oa(l/r)_ was set as 3∶1 using resistors near the battery. Resistance to flow along the uterine branch of the ovarian artery and the uterine artery was modeled by a series of identical resistors (one per implantation site). Resistance to flow into the placenta via the spiral arteries was modeled by low-value, flow-limiting resistors. These resistors were connected separately to a second resistor to ground, simulating exchange within the placenta itself and subsequent flow back to ground (i.e., clearance of blood through the maternal veins). Diffusion across the placenta was modeled by the insertion of a large resistor (20× the value of the dual resistors to ground representing flow into the placenta), between the dual arterial-input resistors. Total maternal blood supply to each placenta I_p,i_ was therefore equal to I_p,oa,i_+I_p,ua,i_.

**Figure 8:**
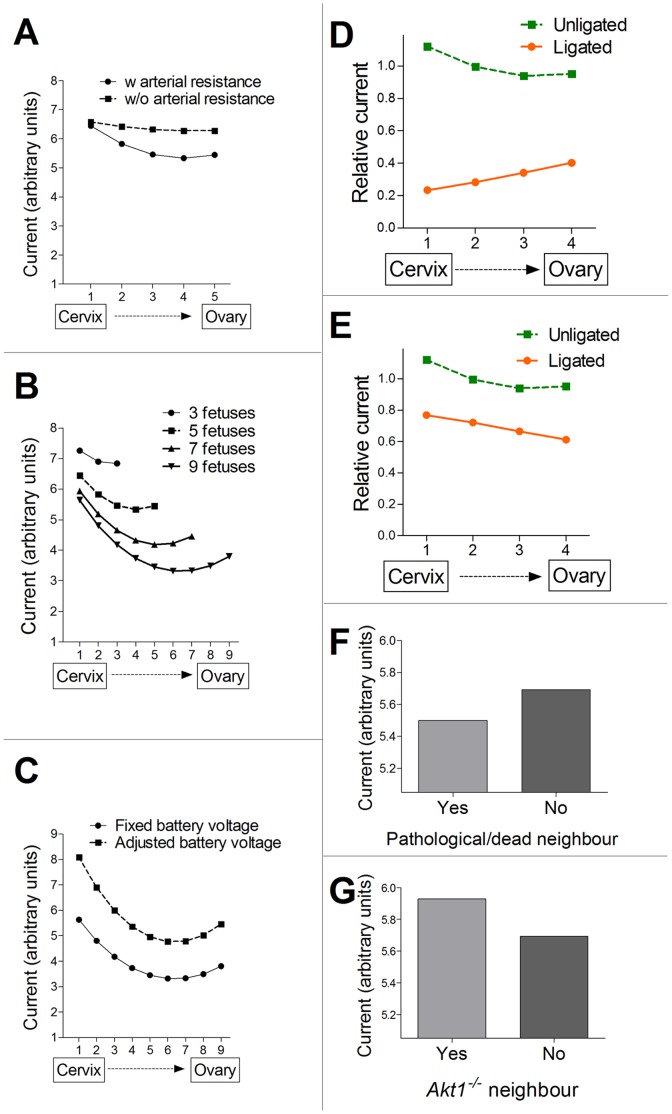
Numerical simulation of placental function in the pregnant mouse reveals a possible hemodynamic basis for the observed ‘position dependence’ and ‘neighbor effects’. (A) Simulation performed for the case of five fetuses in a single uterine horn and a hypothetical case, in which the resistance along the uterine and ovarian arteries was very low. Note the flattening of the U-shaped pattern in the latter case. (B) Simulation of the effect of increasing litter size with a fixed battery voltage V_H_ (no cardiac output compensation)_._ (C) Simulating a case of nine ‘fetuses/placentas’ with adjustment of V_H_ (i.e., adjustment of cardiac output) to maintain the same average I_p_ as in the case of five fetuses per uterine horn. (D) Ligation simulations of the uterine artery showing I_p_ of placentas in the non-ligated and ligated horns, normalized to the average I_p_ of the non-ligated horn. (E) Ligation simulations of the uterine branch of the ovarian artery showing I_p_ of placentas in the non-ligated and ligated horns, normalized to the average I_p_ of the non-ligated horn. (F) Simulation of the placenta of a fetus that spontaneously died during mid pregnancy. The current flowing in normal placentas located closest to the dead fetus is compared to placentas located distant from the dead fetus. The dead fetus was simulated by reduced placental resistance compared to normal placentas. (G) Simulation of *Akt1* ^−/−^ placenta; the average I_p,*Akt1+/+*_ of *Akt1* ^+/+^ having a *Akt1* ^−/−^ neighbor compared with *Akt1* ^+/+^ placentas having only *Akt1* ^+/+^ neighbors. The *Akt1* ^−/−^ placenta was simulated as increased resistance compared to *Akt1* ^+/+^ placentas.

We used the electrical model to simulate maternal perfusion in a normal multi-fetal pregnancy (e.g., five fetuses in each uterine horn; Fig. 8A). These simulations reproduced the reduced perfusion of the central fetal positions. Interestingly, when we simulated a hypothetical case in which the resistance along the uterine and ovarian arteries was very low, this U-shaped pattern flattened, resulting in identical Ip values for all placentas.

Next, we simulated the impact of cardiac adaptation by altering the output voltage of the battery. For a fixed battery voltage, V_H_, simulating a larger litter size resulted in decreased I_p_ for all positions, and particularly for the central positions (Fig. 8B). However, maternal cardiac output can adapt to support the growing fetuses [4]–[6]. To model this phenomenon, separate simulations were performed, in which V_H_ was adjusted to maintain the same average I_p_ as in the case of five fetuses per uterine horn. A 40% increase in V_H_ was required to match the average I_p_ for a litter of 9 fetuses to that of a litter of 5 fetuses (Fig. 8C).

#### Electrical modeling of the impact of arterial ligations

Simulating ligation of either of the two main arteries (i.e., setting either I_ua_ or I_oa_ to zero), resulted in reduced I_p_ for all positions (Fig. 8). The effect on I_p_ of setting I_ua_ = 0 (simulating ligation of the uterine artery) was larger than setting I_oa_ = 0. The largest impact was for lower positions for I_ua_ = 0 and upper positions for I_oa_ = 0 (Fig. 8C, 8D). The pattern in this simulation reproduced the experimental results, with I_p_ increasing gradually in placentas further away from the ligation point.

#### Simulating the ‘neighbor effects’ of genetic or spontaneous pathologies

To simulate the placenta of a fetus that spontaneously died during mid pregnancy, we increased the resistance of the two arterial inputs by a factor of two, while decreasing the resistance to ground by a factor of 10. The rationale for these changes was that in a dead fetus, with little or no maternal-to-placenta exchange, the resistance to flow within the placenta should decrease, while the resistance of the arterial inputs to the placenta should increase, reflecting the expected higher restriction of flow into the placenta. The current flowing in normal ‘placentas’ located closest to the dead fetus was reduced (Fig. 8F), consistent with in vivo BD-ASL MRI and fluorescence experiments.

Finally, we examined the effect of a dead fetus in a model system of pregnancy having only one arterial supply (either the uterine artery or the uterine branch of the ovarian artery). Following the death of a fetus, simulated by altering its resistance as described above, I_p_ decreased for all positions (data not shown). By contrast, for a dual arterial supply, the ‘placentas’ that suffered the largest percentage decrease in current were the nearest neighbors, with less remarkable changes in the rest of the ’placentas’ in that uterine horn.

It was previously reported that *Akt1^−/−^* placentas have a two-fold lower maternal blood volume fraction and a reduced placental circulatory bed (i.e., placental labyrinth, including both maternal and fetal blood spaces) [18], [35]. Accordingly, using the electrical model, an *Akt1^−/−^* placenta was simulated by two-fold higher resistance. As expected, I_p,*Akt1−/−*_ was lower than that of *Akt1^+/+^*. Remarkably, the average I_p,*Akt1+/+*_ with an *Akt1^−/−^* neighbor was found to be 5% higher than that of *Akt1^+/+^* placentas having only *Akt1^+/+^* neighbors (Fig. 8G).

## Discussion

During the course of pregnancy, coordinated growth and remodeling of the maternal vasculature, as well as adaptation in the maternal cardiovascular system, are essential for meeting the requirements of the growing fetus(es), and responding to challenges of physiologic and/or pathologic conditions. In litter-bearing mammals, these qualities are particularly important, as these species must support multiple fetuses within one uterus. The unique functional hemodynamic basis of the maternal vasculature system and the related in utero positional- and inter-fetal dependent effects are the focus of this study.

Using a multimodal, longitudinal imaging strategy, which included BD-ASL MRI, intra-vital microscopy, and fluorescence imaging, we were able to characterize the functional anatomy of the mouse pregnancy and the patterns of maternal-to-fetal water exchange, as well as the maternal blood volume, in placentas during normal pregnancy and pregnancies complicated by spontaneous, surgical and genetic interventions. Hemodynamics of the maternal blood flow was simulated numerically using an electrical model, which was structured based on the physiological findings. This model helps to explain the underlying hemodynamic basis for the observed arterial blood supply to the uterus and placentas in multi-fetal pregnancy, and allows us to explore extreme conditions that may not be accessible experimentally.

Previous studies reported that pregnancy in rodents is supported by bi-directional flow of blood within a uterine arterial loop [15], [16]. This anatomical description is inconsistent with the data reported here. We show herein that in the mouse, each placenta is supplied in parallel by dual arterial inflow originating from both of the main arteries, the uterine artery and the uterine branch of the ovarian artery, resulting in mosaic irrigation fields within each placenta. Thus, in the time frame of T_1_ relaxation of water (i.e., seconds), the two arterial inputs do not mix prior to their entry into the placenta. Although we do not exclude the presence or functionality of uterine arterial anastomosis, we provide here direct experimental evidence that such anastomosis cannot be the primary mechanism for maternal arterial input to the placenta in mouse pregnancy.

The ratio of the blood supplied by the two feeding arteries varied with litter size within the uterine horn. Our results indicate that as the number of fetuses/placentas increased from 4 to 6 per uterine horn, the relative contribution of the uterine artery increased. Therefore, it appears that large-scale vascular remodeling can occur in order to meet the demands of individual pregnancies. In woman, the uterus is mainly supplied with blood by the uterine arteries, while the contribution of the ovarian arteries is less significant [41]. Interestingly, pregnancy can be established in women treated for postpartum hemorrhage and uterine myomas by either uterine artery embolization or arterial ligation, providing a human example of this remodeling of collateral blood flow [42].

As expected, an increased number of fetuses per uterine horn was associated with reduced fetal body weight. Such systemic effects could be attributed to a drop in maternal blood pressure associated with the increased fetal load, competition among fetuses for a limited amount of space, as well as gas and nutrient transport insufficiency [43]. One mechanism of compensating for the reduced placenta inflow in large litters is an increase in cardiac output during pregnancy, as was shown previously in many species [4]–[6], [44].

A dual arterial supply to each placenta provides robustness to the maternal support of pregnancy. As mentioned above, fetuses located at the edges of the uterine horn appear to enjoy more favorable growth conditions vis-à-vis blood flow and volume. However, according to our data, we speculate that those fetuses located in the middle of the uterine horn may be less susceptible to damage when one of the maternal arteries is compromised. Therefore, the dual arterial design of the placenta may confer a growth advantage to the fetuses at both edges of the horn in unperturbed pregnancy, while providing a survival advantage to fetuses located in the middle of the uterine horn in cases of severe maternal arterial damage.

The complex design of the maternal circulation can result in interesting hemodynamic effects on fetal development. In particular, we report here that beyond the direct effect on the affected placenta, fetal-specific pathological processes, such as spontaneous fetal growth retardation or death, or even a genetic difference, occurring in an adjacent placenta, can significantly alter maternal blood inflow in the placenta of an otherwise healthy fetus. Such an effect should be added to previously reported molecular mechanisms (e.g., the local diffusion of hormones affecting gender-related changes) describing the influence of a fetus on adjacent siblings [45]–[47].

Interestingly, we found that the placentas of fetuses that were located closest to the pathological fetus also exhibited significantly lower maternal-to-placental exchange and maternal blood volume, compared with that expected based upon their position along the uterine horn. Electrical simulation of a pathological/dead fetus in a pregnancy supported by a single arterial supply (either the uterine artery or the ovarian artery) resulted in a significant decrease in the current flowing in all of the placentas located downstream of the pathological/dead one, with a small systemic effect in placentas located upstream of it. Such simulations highlighted the robustness of the dual arterial blood supply to the uterine horn, which is able to compensate for the loss of a single fetus and sustain blood flow to the remaining siblings.

To evaluate the effects of genetic heterogeneity within a single litter on possible in utero environmental factors, an *Akt1* model was employed. *Akt1^+/−^* female mice were mated with *Akt1^+/−^* males in order to generate fetuses of all possible genetic backgrounds within the same pregnancy, with the same maternal genetic background. This allowed us to investigate inter-fetal effects without confounding effects related to maternal genotype. *Akt1^−/−^* placentas were reported previously to have reduced vasculature and smaller areas of exchange within the labyrinth [18], [35]. Furthermore, the *Akt1^−/−^* fetuses were shown to be small, with defective vasculature, suggesting *Akt1^−/−^* as a genetic model for human IUGR. Consistent with these studies, we demonstrated that *Akt1^−/−^* placentas showed reduced maternal-to-placental exchange and maternal blood volume compared to *Akt1^+/+^* placentas. Interestingly, we found that *Akt1^+/+^* placentas positioned adjacent to *Akt1^−/−^* placentas displayed a significant increase in both their BD-ASL MRI values and ex vivo fluorescence signals compared to *Akt1* ^+/+^ placentas situated near other normal placentas. This finding suggests that the presence of a small, IUGR-like fetus with placental insufficiency might actually improve placental perfusion in its wild-type neighbouring siblings. In accordance, the hemodynamic effects of *Akt1^−/−^* placentas on the neighboring placenta could be simulated using the electrical model by increasing their resistance by a factor of 2. Furthermore, since metabolism and nutrient demand of *Akt1^−/−^* fetuses may be reduced, it is reasonable to speculate that adjacent *Akt1* ^+/+^ fetuses would benefit, adding a potential metabolic component to the complexity of the reported MR hemodynamic measurements.

The hemodynamic effects of genetic or spontaneous pathologies show that in addition to the systemic effects, which impact the litter as whole, maternal arterial supply might be different for different fetuses in the uterus, such that maternal supply to one placenta can be directly affected by the condition of the neighboring placentas. Moreover, these findings provide an example of how fetal growth must be accommodated by corresponding adaptation of the maternal vasculature. An important implication of this finding is that variability of phenotypes in genetic mouse models could arise from the location and order of fetuses of different genotypes along the uterine horn. As a consequence, in future studies that examine phenotypes of fetuses/pups of different genotypes, confounding local and environmental in utero effects should be considered, both for each individual fetus, and in combination with the genotype of its adjacent siblings. Furthermore, since Akt was found to play a critical role in angiogenesis, cell survival and differentiation, and metabolism, future studies should further elucidate the possible effects of the mother genotype on placental development and function in combination with the in utero environment.

In summary, the current study demonstrated the unique functional architecture of the dual arterial supply, which supports robust maternal circulation in the mouse pregnancy, thus sustaining large-litter pregnancies. The experiments revealed complex intra-uterine environmental interactions that exert a significant impact on fetal development and placental function. In particular, we discovered distinctive hemodynamic effects of fetal position on placental perfusion, and hemodynamic effects of genetic or spontaneous pathologies on adjacent neighbouring siblings. The presence of the genetically defective placenta of an *Akt1^−/−^* fetus was associated with increased maternal-to-fetal exchange and increased maternal blood volume in the placenta of its nearest *Akt1^+/+^* siblings. Conversely, the presence of a spontaneously pathological/dead fetus in utero was associated with significantly reduced maternal-to-fetal exchange and reduced maternal blood volume in the placenta of its nearest siblings. These findings highlight the complexity of prenatal development in litter-bearing mammals. Remarkably, the dual arterial supply is also conserved in mammals bearing a single offspring, including primates [48].

## Supporting Information

Figure S1
**Calibration between ex vivo fluorescence signal and the placental maternal blood volume (PBVm).** To correlate the normalized placental fluorescence signal with the placental maternal blood volume (PBVm), a calibration experiment was performed. Dextran-FITC was intravenously injected into female ICR mice at E17.5 (n = 6), and blood samples were drawn from each mouse 10 min later. The fluorescence signals of fixed blood volumes (0.01, 0.03, 0.05, 0.08, 0.1, 0.15 and 0.3 ml) were measured, and were normalized, in each mouse, to the signal in the uterine artery. Ex vivo fluorescence signal intensities were positively correlated to blood volume (r = 0.97, *P*<0.0001), thereby establishing a calibration equation for calculating placental maternal blood volume from normalized fluorescence signal: 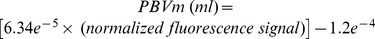

(TIF)

Figure S2 **Correlation between peak BD-ASL values and the relative location of fetuses along the uterine horn in** ***PKBAkt1^−/−^*** **pregnant mice (E17.5) carry fetuses of different genotype.** Peak BD-ASL values showed significant positive correlation with the position of placentas along the uterine horn (r = 0.63, *P*<0.0001). Note that fetal implantation location was randomly distributed along the uterine horn for all genotypes (*P* = 0.8941).
(TIF)

Movie S1 **Intravital microscopy of surgically-exposed intact uterine horn following intravenous administration of fluorescent FITC-dextran.**
(MPG)

Movie S2 **Intravital microscopy of surgically-exposed uterine horn following ligation of the uterine artery and intravenous administration of fluorescent FITC-dextran.**
(MPG)

Movie S3 **Intravital microscopy of surgically-exposed uterine horn following ligation of the uterine branch of the ovarian artery and intravenous administration of fluorescent FITC-dextran.**
(MPG)
