# Supplementary figures and images for: The Hemodynamic Basis for Positional- and Inter-Fetal Dependent Effects in Dual Arterial Supply of Mouse Pregnancies
Source: PLoS One. 2012 Dec 20;7(12):e52273. doi: 10.1371/journal.pone.0052273 (PMC3527527; doi:10.1371/journal.pone.0052273)

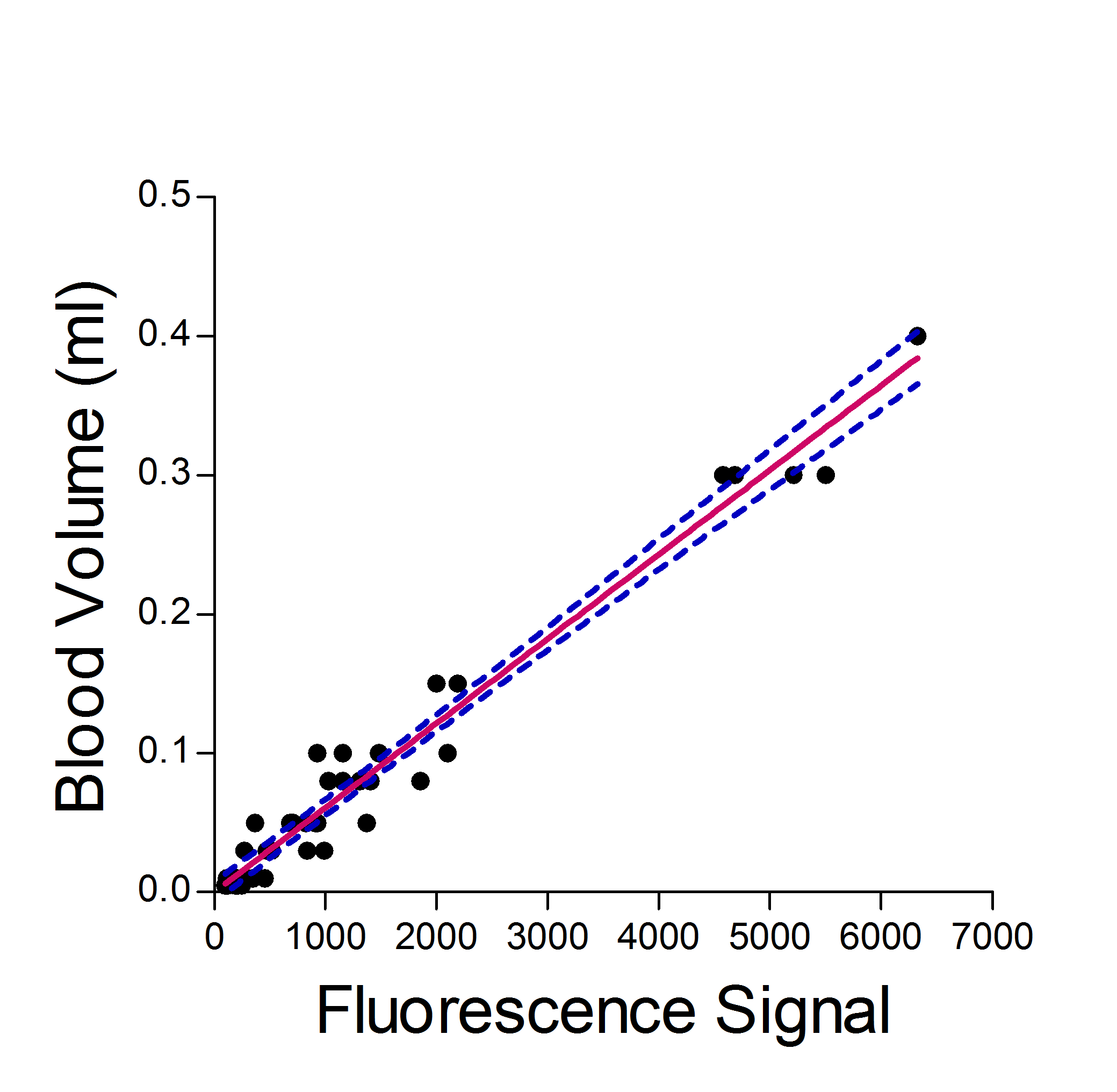

Supplement: Figure S1 — Calibration between ex vivo fluorescence signal and the placental maternal blood volume (PBVm). To correlate the normalized placental fluorescence signal with the placental maternal blood volume (PBVm), a calibration experiment was performed. Dextran-FITC was intravenously injected into female ICR mice at E17.5 (n = 6), and blood samples were drawn from each mouse 10 min later. The fluorescence signals of fixed blood volumes (0.01, 0.03, 0.05, 0.08, 0.1, 0.15 and 0.3 ml) were measured, and were normalized, in each mouse, to the signal in the uterine artery. Ex vivo fluorescence signal intensities were positively correlated to blood volume (r = 0.97, P<0.0001), thereby establishing a calibration equation for calculating placental maternal blood volume from normalized fluorescence signal: (TIF) [file pone.0052273.s001.tif]

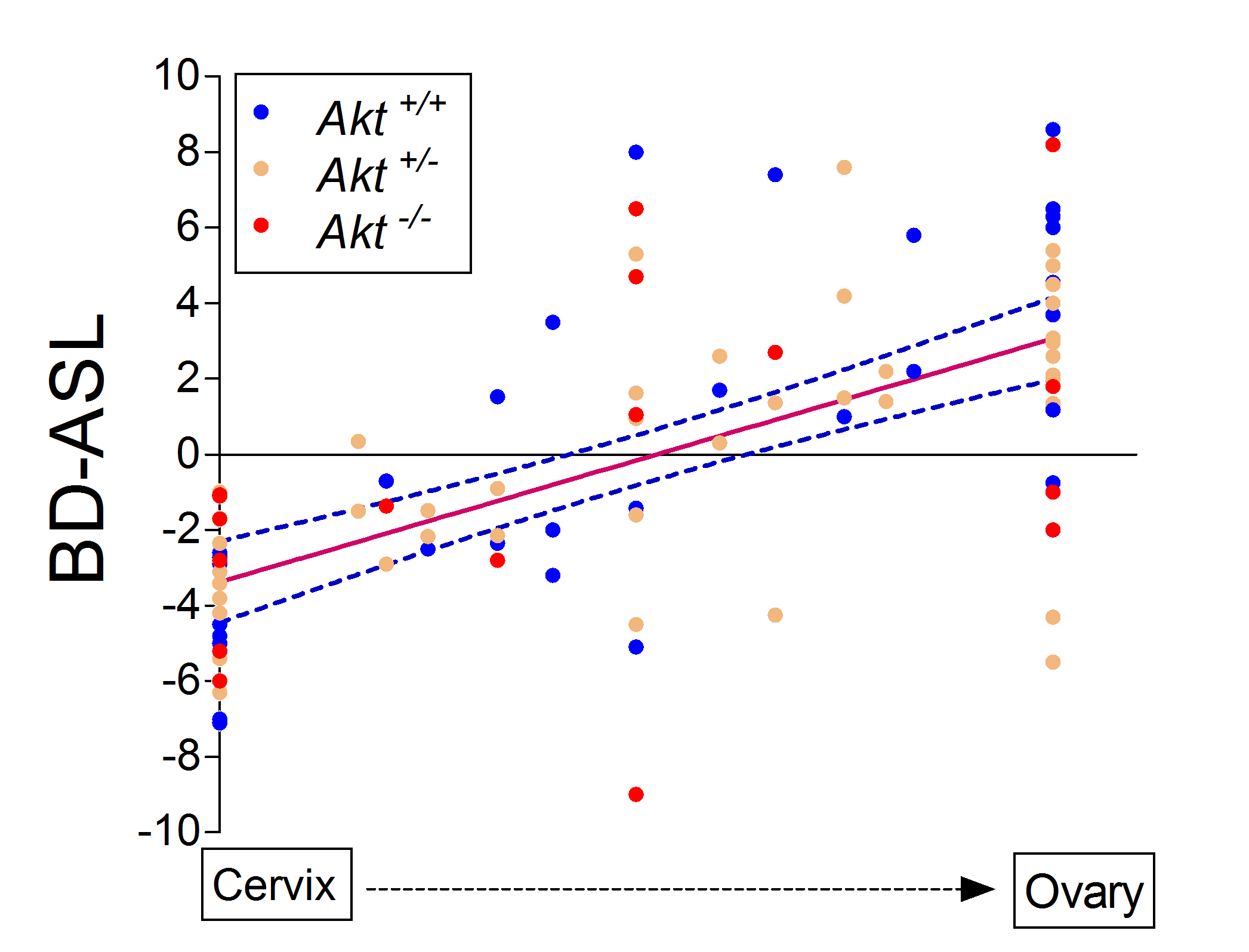

Supplement: Figure S2 — Correlation between peak BD-ASL values and the relative location of fetuses along the uterine horn in PKBAkt1−/− pregnant mice (E17.5) carry fetuses of different genotype. Peak BD-ASL values showed significant positive correlation with the position of placentas along the uterine horn (r = 0.63, P<0.0001). Note that fetal implantation location was randomly distributed along the uterine horn for all genotypes (P = 0.8941). (TIF) [file pone.0052273.s002.tif]
